# Cryo-Electrohydrodynamic Jetting of Aqueous Silk Fibroin Solutions

**DOI:** 10.1021/acsbiomaterials.3c00851

**Published:** 2023-11-21

**Authors:** Ander Reizabal, Paula G. Saiz, Simon Luposchainsky, Ievgenii Liashenko, DeShea Chasko, Senentxu Lanceros-Méndez, Gabriella Lindberg, Paul D. Dalton

**Affiliations:** † Phil and Penny Knight Campus for Accelerating Scientific Impact, University of Oregon, 1505 Franklin Boulevard, Eugene 97403, Oregon, United States; ‡ BCMaterials, Basque Center for Materials, Applications and Nanostructures, Bldg. Martina Casiano, UPV/EHU Science Park, Barrio Sarriena s/n, 48940 Leioa, Spain; § Macromolecular Chemistry Group (LABQUIMAC), Department of Physical Chemistry, Faculty of Science and Technology, University of the Basque Country (UPV/EHU), Barrio Sarriena s/n, E-48940 Leioa, Spain; ∥ Ikerbasque, Basque Foundation for Science, Bilbao 48009, Spain

**Keywords:** additive manufacturing, near-field electrospinning, cryogenic, electrowriting, biomaterials

## Abstract

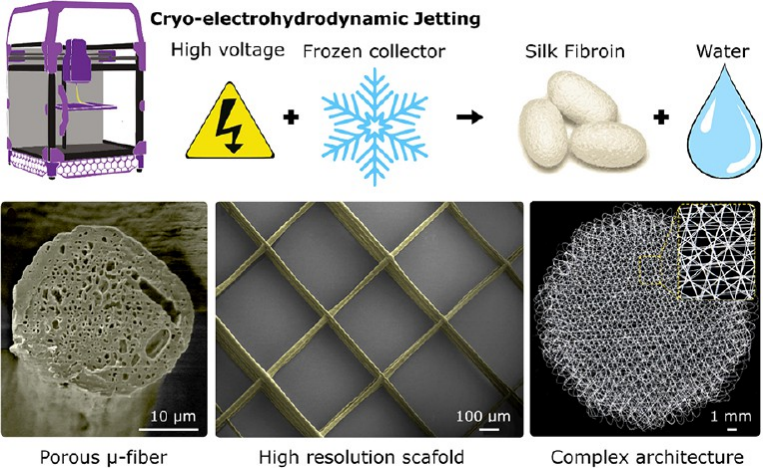

The incorporation of 3D-printing principles with electrohydrodynamic (EHD) jetting provides a harmonious balance between resolution and processing speed, allowing for the creation of high-resolution centimeter-scale constructs. Typically, EHD jetting of polymer melts offers the advantage of rapid solidification, while processing polymer solutions requires solvent evaporation to transition into solid fibers, creating challenges for reliable printing. This study navigates a hybrid approach aimed at minimizing printing instabilities by combining viscous solutions and achieving rapid solidification through freezing. Our method introduces and fully describes a modified open-source 3D printer equipped with a frozen collector that operates at −35 °C. As a proof of concept, highly concentrated silk fibroin aqueous solutions are processed into stable micrometer scale jets, which rapidly solidify upon contact with the frozen collector. This results in the formation of uniform microfibers characterized by an average diameter of 27 ± 5 μm, a textured surface, and porous internal channels. The absence of instabilities and the notably fast direct writing speed of 42 mm·s^–1^ enable precise, fast, and reliable deposition of these fibers into porous constructs spanning several centimeters. The effectiveness of this approach is demonstrated by the consistent production of biologically relevant scaffolds that can be customized with varying pore sizes and shapes. The achieved degree of control over micrometric jet solidification and deposition dynamics represents a significant advancement in EHD jetting, particularly within the domain of aqueous polymer solutions, offering new opportunities for the development of intricate and functional biological structures.

## 1 Introduction

Porous 3D-printed scaffolds are extensively used to support and guide cell growth.^1^ Among the techniques for creating these structures, melt electrowriting (MEW), stands out due to its ability to produce micrometer-sized fibers and systematically stack them to craft specific 3D scaffolds.^2^ As an electrohydrodynamic (EHD) processing technology,^3^ MEW involves the extrusion of molten polymers by leveraging forces of electrical fields. Molten polymers, in particular, provide several processing advantages, such as high viscosity, limited electrical conductivity, and rapid solidification. Together, these characteristics minimize system instabilities,^4^ ensuring stable processing even over extended periods.^5^ Additionally, MEW eliminates the need for solvents, simplifying equipment requirements and preventing the presence of toxic residues in the final materials. Furthermore, melt processing is a known strategy to safely fabricate biomedical devices,^6^ thus, the utilization of polymers with a history of medical applications aids in the clinical translation of biomaterials.^7^

Compared to other extrusion-based 3D-printing technologies, MEW strikes an excellent balance between the extrudate diameter and placement resolution.^8^ MEW enables precise fiber deposition with diameters as small as 0.8 μm,^9^ which is a fiber diameter reduction of around 2 orders of magnitude compared to the smallest extrudate from traditional melt extrusion 3D-printing systems.^10^ MEW achieves such small diameters by adopting flow rates ranging from 0.5 to 20 μL/h,^11^ and allows the creation of anatomically relevant scaffolds,^12^ all while achieving printing speeds of 1500 mm/min.^11^ These characteristics represent a significant leap in scale compared to other high-resolution 3D-printing technologies.^13,14^ Furthermore, MEW does not require expensive instrumentation,^11^ can be easily upgraded for high-throughput applications,^15^ and is suitable for processing active materials.^16,17^ This precision and versatility not only make MEW valuable for tissue engineering but also have broad utility in fields where microscale device fabrication is required.^18^

Despite its promising features, MEW faces certain challenges as a relatively recent technology. These include demanding thermal processing requirements and the utilization of thermoplastic materials that often lack inherent integrated biological activity.^19^ In light of these challenges, EHD jetting of aqueous solutions, or aqueous electrowriting, emerges as a complementary alternative for scaffold fabrication.^3^ This approach, based on similar physical principles to MEW, enables the use of biobased polymers while circumventing the toxicity concerns associated with organic solvents.^3^ Although EHD spinning, i.e., electrospinning, has explored the use of aqueous solutions, it typically requires collector distances on the order of many centimeters to achieve adequate solvent evaporation. Additionally, EHD instabilities linked to electrospinning significantly impact fiber deposition leading to random fiber accumulation.^3,20^ Various studies have endeavored to mitigate EHD instabilities in aqueous solutions for precise fiber positioning and reliable jet solidification.^3^ These efforts encompass the use of viscous solutions, such as silk fibroin (SF)/polyethylene glycol (PEO: 600,000–1,000,000 Da) hydrogels,^21^ highly concentrated silk aqueous solutions (∼30 wt %/wt),^22^ and fast solidification systems like UV photo-cross-linking or liquid nitrogen-driven cryogenic systems.^23,24^ However, achieving the same level of jet stability as MEW without resorting to complex systems, undesirable additives, and organic solvents remains a significant challenge.^3,22^

In our study, we attain a high degree of control over jetting by combining the printing stability provided by viscous water solutions, in conjunction with a system that enables high printing speeds and accuracy, and collector temperatures down to −35 °C. SF, a protein extracted from the silkworm cocoon, was selected as the proof-of-concept polymer due to its versatility,^25,26^ including its wide use in biomedical applications,^27−29^ and specific physical-chemical characteristics, such as water solubility, ability to become water-stable through secondary treatments,^30^ and unique mechanical properties. The control of both solution molecular entanglements and solidification dynamics by increasing the SF concentration, and freezing the jet upon contact with the collector, proved crucial in achieving stable jetting with favorable printing outcomes.^3,22^ Furthermore, the used 3D system to conduct the research is compatible with MEWron project, which aims to make the MEW and EHD processing more accessible.^11^ The design and implementation of all the 3D system components are fully available in the manuscript.

## 2 Materials and Methods

### 2.1 Materials for SF Solution

*Bombyx mori* cocoons were purchased from Treenway Silks (Colorado, USA). Sodium carbonate (Na_2_CO_3_), lithium bromide (LiBr), ethanol (EtOH), dialysis tubes with a diameter of 3 cm and molecular weight cutoff of 3500 Da, and % polyethylene oxide (20,000 Da) were acquired from Fisher Scientific.

### 2.2 Silk Solution Formulation

**Figure 1:**
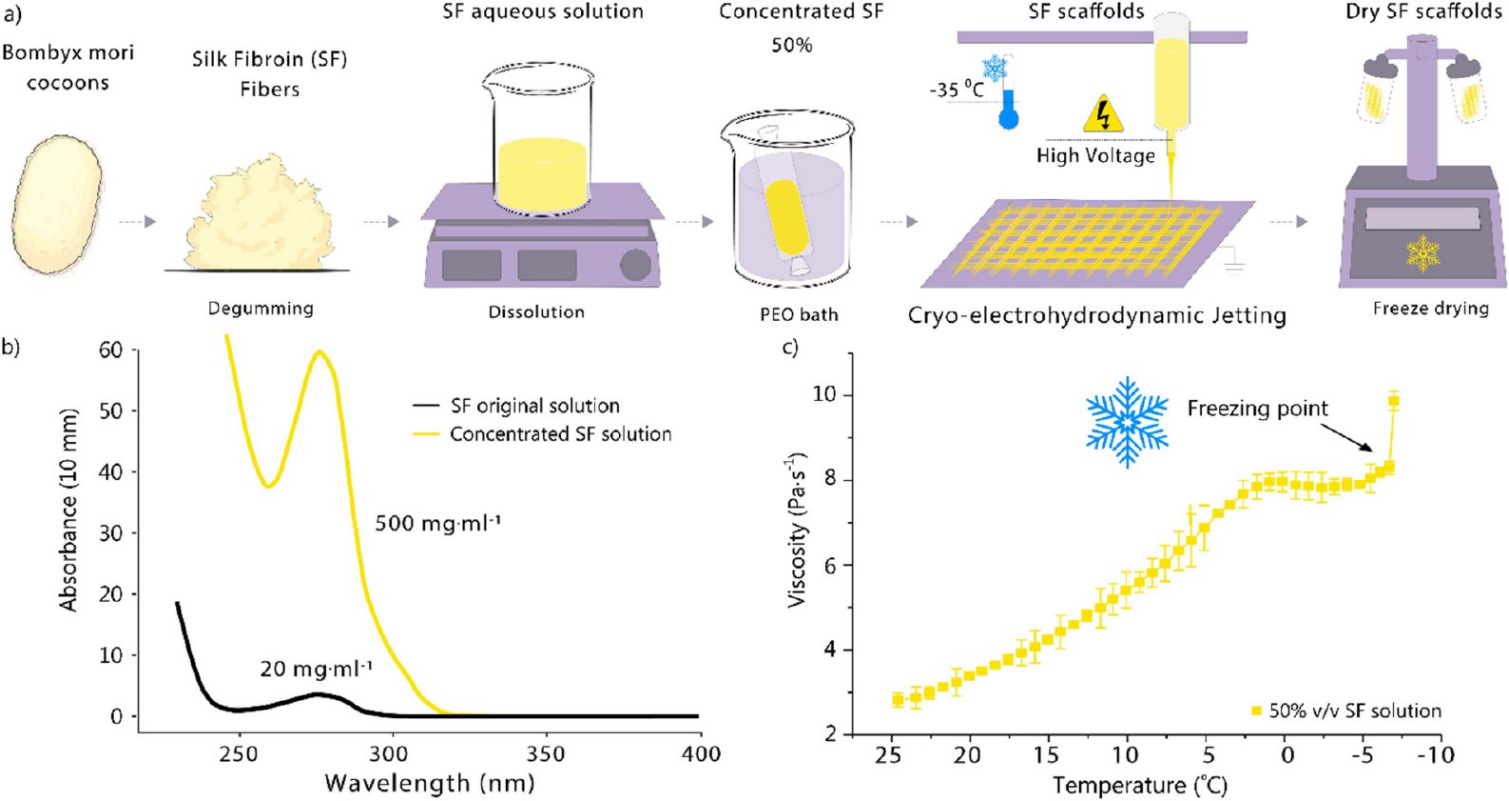
(a) Schematic of highly viscous silk fibroin (SF) aqueous solution processing by cryo-electrohydrodynamic jetting. The silk is initially sourced from silkworm cocoons, subjected to degumming to extract the SF, and dissolved in a LiBr solution. Following a series of dialysis steps to remove dissolved salts and concentrate the solution, a 50% viscous SF aqueous solution is achieved. This concentrated solution is directly utilized in the cryo-electrohydrodynamic jetting system to produce porous and fibrillar scaffolds. To eliminate the water content within the scaffolds, they undergo a freeze-drying process. (b) UV–vis spectra of SF aqueous solution before and after polyethylene glycol (PEG: 20,000 Da) concentration. The obtained final concentration is of 500 mg·mL^−1^ (50% v/v). (b) Concentrated SF solution viscosity as a function of temperature. The solution freezes at −8 °C.

A scheme of the following experimental procedure can be found in Figure 1a. SF was extracted from *Bombyx mori* cocoons. For that, cocoons were cleaned, cut into small ∼1 cm^2^ pieces, and immersed twice at 80–85 °C in an aqueous bath for 10 min in a 3.7 mM Na_2_CO_3_ solution [silk-to-liquid ratio 1:10 (wt (g):v (mL)] to remove the non-necessary silk sericin (degumming). The remaining SF strands were vigorously cleaned in distilled water and finally dried at room temperature (RT).

To obtain the SF solution, degummed silk fibers were dissolved in a 9.3 M LiBr aqueous solution at 60 °C (SF-to-liquid ratio 1:4 g:mL). The obtained tinted yellow solution was filtered with 80 μm mesh to remove any solid impurities and dialyzed in a 3500 Da cellulose dialysis tube against distilled water. The water bath was replaced at least three times per day until constant conductivity values were measured on dialysis water, indicative of the salts’ maximum removal.

During EHD jetting, molecular entanglements play a critical role in reducing instabilities and preventing jet breakage.^31^ Therefore, highly viscous SF solutions were prepared by concentrating a silk aqueous solution by dialysis against a 20% PEG (20,000 Da) solution. After 2–3 days, a target concentration of 50% (v/v) was achieved (Figure 1b). At RT, such solutions exhibit a viscosity of 3 Pa·s at a shear rate of 10 s^–1^, which gradually increases with decreasing temperature, reaching 8 Pa·s at 2 °C (Figure 1c). The viscosity remained stable down to −8 °C when the solution freezes. The obtained SF aqueous solution was stored for 1 week at 4 °C.

### 2.3 Cryo-EHD Jetting

**Figure 2:**
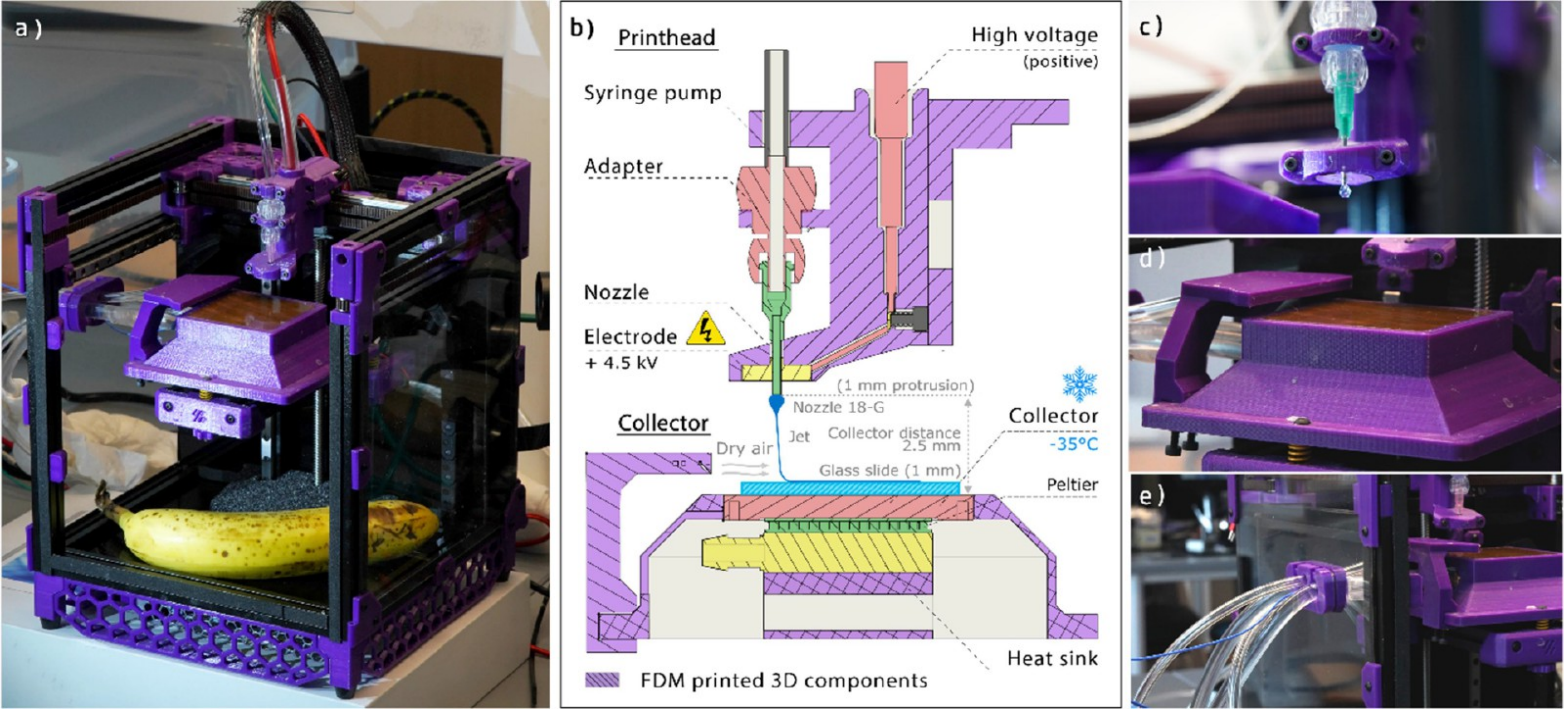
(a) Overview of the custom-made cryo-EHD jetting system. (b) Cross-section of the custom 3D-printed printhead and collector and diagram of cryo-EHD jetting system setup. An 18-gauge nozzle is positioned in contact with a copper toroid, which is subjected to a voltage of 4.5 kV. The nozzle protrudes 1 mm from the toroid and is situated 2.5 mm away from the glass collector. The glass slice, measuring 1 mm in thickness, is positioned above the frozen collector, which is temperature-controlled using a thermoelectric device set at −35 °C. The collector is grounded to create an electric field between the nozzle and the substrate. A dry air environment avoids ice crystal formation during the printing process. (d) Details of the printer’s main components: (c) printhead, (d) cryo-collector and dry air blow system, and (e) collector connection to the chiller and dry air system.

To achieve 3D architectures from aqueous solutions, a Voron 0.1 fused filament fabrication (FFF) system was customized to incorporate an electrically isolated frozen collector and a syringe holder (Figure 2a). The choice of Voron printers was deliberate, stemming from their high printing quality, strong community support, and adherence to open-source philosophy. We specifically opted for Version 0.1 of the Voron printer due to its compact size, printing stability, and affordability, typically falling within the price range of 600–800 USD. Moreover, this conversion approach can be extrapolated to other Voron printer models, which may offer additional functionalities such as a larger printing space or the ability to perform two-headed simultaneous printing.

Before commencing the conversion to the cryo-EHD jetting system, a fully functional and well-calibrated Voron 0.1 printer is required.^32^ It is important to note that these printers are assembled by the user and involve FFF printing of various components, as well as the handling of electrical wiring. Consequently, it is imperative that users meticulously follow the official manual while exercising caution and prioritizing safety throughout the assembly process.^11,33^

The original printhead was substituted with a customized FFF design capable of housing a Tuohy-Borst adapter (from Qosina, model FLO 30), which enables swappable connection of different diameter tubes with Luer lock nozzles (Figure 2b,c). The selected setup consists of an 18-gauge blunt nozzle (0.8 mm ID, 1.28 mm OD, 1.25 cm length, from BSTEAN) connected through a silicone tube [0.8 mm inner diameter (ID) and 1.6 outer diameter (OD), from Quickun] to a syringe pump (from World Precision Instruments, model AL-300). This enables the dispensed solution volume control under a variety of flow rates, including the common ones required for EHD jetting and MEW.^11^ Besides, the FFF printhead enables the nozzle contact with a copper square toroid (5 mm height, 20 mm OD, and 1 mm ID) connected to a high-voltage power supply (from Heinzinger, model LNC 10000–2 pos). A nozzle protrusion of 1 mm measured from the bottom face of the printhead to the tip of the nozzle was manually set (Figure 2b,c).

Given the low evaporation rate of water and its implications on fiber solidification, cooling the substrate was deemed the most viable option to solidify the jet. There are several approaches to generate frozen substrates,^3,22^ and here we used a Peltier device due to its capacity to precisely control temperature based on an applied current. For the collector, a potted Peltier (TE technology, model HP-127–1.4–1.15–71) was placed in contact with a custom-made copper block (5 mm thick and 60 mm square), which served as the printing collector (Figure 2b,d). The Peltier performance was controlled through a DC Power Supply (RS310P). To remove the exceed of heat, the hot side of the Peltier was placed in contact with a liquid cooling block (heatsink −40 mm × 40 mm and 10 mm high), connected to a chiller (Fisher Scientific Isotemp 250LCU) loaded with glycerol–water (50/50) recirculating liquid at −5 °C (Figure 2e). The setup reached stable temperatures of −35 °C in the collector surface (Figure S1a).

To prevent the formation of ice crystals on the frozen collector, the 3D printer was fully enclosed and sealed with foam tape on corners, and a positive pressure dry air stream was added on the inside (Figure 2e). Two different airlines were included, the first one oriented toward the collector and the second one toward the printer’s floor. Additionally, a small access port was installed into the main door, to limit moisture ingress to the collector during sample collection and preparation. The setup achieves relative humidity values below 5% at RT after 20 min of stabilization (Figure S1b).

The 3D printer movement is controlled via G-code as numerical control and allows for printing speeds up to 70 mm·s^–1^. Further modification details about the modified open-source 3D printer, including the configuration overview (Figure S1c), detailed cryo-EHD jetting system components (Figure S2), video summarizing the Voron3D printer modifications (Video S1), and computer-aided design files, can be found in the Supporting files.

For all experiments, a 2.5 mm collector distance was used and a voltage of +4.5 kV was applied to the nozzle while the collector was earthed. A 1 mm thick glass slide was placed upon the collector and the collector speed was adjusted between 4.2 and 50 mm·s^–1^, depending on the specific experiment. To maintain a continuous and steady solution supply under various conditions, the flow rate was between 40 and 100 μL·h^–1^. The SF solution was kept at RT before printing and was left in the freezer after solidification. Additional experimental details can be found in Table 1.

**Table 1:** Default Processing Conditions for SF Aqueous Solutions

variable	value
SF concentration	50 mg·mL^–1^
nozzle inner diameter	800 μm
voltage	4.5 kV
working distance	2.5 mm
flow rate	40 μL/h
room temperature	22 °C
relative humidity	2–5%
collector temperature	–35 °C
printing speed	2500 mm·min^–1^
drying approach	freeze-drying
stabilization	EtOH

### 2.4 SF Scaffold Stabilization

The processed scaffolds were rapidly translated to a −20 °C freezer. To prevent melting of the collected material, a frozen aluminum plate was used as a support for scaffold transfer to and from the freezer. The SF fiber scaffolds were lyophilized in a FreeZone 4.5 L–50 °C Benchtop Freeze-Dryer from Labconco, US. To prevent SF fiber solubility, the lyophilized scaffolds were dipped in 80% ethanol (EtOH) and dried at RT.^30^ This process is known for its ability to promote SF cross-linking and maintain SF structure shape intact.

### 2.5 EHD System, Solutions, and Scaffolds Characterization

Temperature and relative humidity data were collected from a Tempy wireless cloud-connected sensor. Viscosity measurements were performed on an HR-2 Discovery Hybrid rheometer from TA Instruments. Optical microscopy images were obtained from a VHX-7000 Keyence microscope and a Sony Alpha 7 III full-frame camera. Scaffold morphology was evaluated by scanning electron microscopy (SEM) with an Apreo 2 SEM from Thermo Fisher. Fourier-transform infrared (FTIR) spectroscopy in the attenuated total reflection (ATR) mode was performed at RT in a Jasco FT/IR-6100 from 4000 to 600 cm^–1^ collected with 64 scans at a resolution of 4 cm^–1^. The secondary structure of the SF samples was obtained from amide I, C=O stretching band (1700–1600 cm^–1^) deconvolution of ATR/FTIR data by OriginPro 8.1 software (OriginLab, Northampton) following a previously reported procedure.^34^ Briefly, the amide I peak was deconvoluted into 12–14 peaks, and each one was assigned to a specific secondary structure as a function of the peak maximum: side chains (SC) between 1580 and 1609 cm^–1^; β-sheets (B) between 1609 and 1631 cm^–1^ and 1691 and 1711 cm^–1^; random coils (RC) between 1631 and 1658 cm^–1^; α-helix (A) between 1658 and 1666 cm^–1^ and turns (T) between 1666 and 1691 cm^–1^. Finally, the areas below the peaks were added as a function of the assigned secondary structures.

### 2.6 Materials for Cell Culture and Biological Evaluation

Gibco αMEM nucleosides glutaMAX, Gibco DMEM hi-glucose glutamax media, bovine serum albumin (BSA), AlamarBlue, Gibco 4-(2-hydroxyethyl)-1-piperazineethanesulfonic acid (HEPES), Gibco nonessential amino acids (NEAA), penicillin-streptomycin (10.000 U mL^–1^), Gibco fetal bovine serum (FBS) and goat-antimouse (GaM) secondary antibody (Alexa Fluor 488) were supplied by Fisher Scientific. Glycine, gelatin (porcine skin, type A, gel strength 300), allyl glycidyl ether (≥99%), Β-glycerophosphate, proline, l-ascorbic acid-2-phosphate sesquimagnesium salt (AsAp), dexamethasone, acetic acid (≥99.8%), hydrochloric acid (HCl) (37%), sodium chloride (NaCl) (≥99%), sodium hydroxide (NaOH) (>98%), and lithium phenyl-2,4,6-trimethylbenzoylphosphinate were obtained from Sigma-Aldrich (Merk). Dialysis tubing cellulose membrane (MWCO 1000 Da), dithiothreitol, and molecular probes 6-diamidino-2-phenylindole (DAPI) and rhodamine-phalloidin for F-actin were sourced from VWR. Primary antibodies for anti-osteopontin (mouse) and anti-collagen type I antibody (rabbit) were purchased from Developmental Studies Hybridoma Bank. Donkey-antirabbit (DaR) secondary antibody (Alexa Fluor 594) was purchased from Abcam.

### 2.7 Cytotoxicity Assay

Extract cytotoxicity of SF and poly(ε-caprolactone) (PCL) scaffolds and raw SF films was tested following the International Organization for Standardization tests for in vitro cytotoxicity (ISO 10993-5:2009) to evaluate the effect of the material preparation and the fabrication process individually. Synthetic nitrile rubber films and PCL scaffolds were used as controls. All test materials were sterilized in 70% ethanol for 30 min and washed in phosphate-buffered saline (PBS) three times before use. The test extracts were prepared by incubating the materials (SF film and synthetic rubber: 6 mg/mL, SF and PCL scaffolds: 9 cm^2^/mL) in expansion medium [Gibco DMEM high glucose glutaMAX supplemented with 0.4 mM l-proline, 10 mM HEPES, 0.1 mM NEAA, 100 U/mL penicillin, 0.1 mg/mL streptomycin, 0.1 mM AsAp and 10% (v/v) FBS] for 24 h at 37 °C in a humidified air incubator (5% CO_2_/95% air).

Human dermal fibroblasts (hDFs: ZenBio, Durham, USA) were seeded in 96-well plates (4.000 cells/well, passage 4), cultured in the previously described expansion medium, and incubated at 37 °C in a humidified air incubator (5% CO_2_/95% air). After 48 h culture, the medium was removed and replaced with 300 μL of PBS (blank control, data not shown), expansion medium (baseline), or expansion medium with ethanol (pos. controls), or material extracts of either nitrile rubber (neg. control), PCL (neg. control), SF film, or SF scaffold. AlamarBlue was used to quantify the metabolic activity, indicative of the total cell number, as per the manufacturer’s description. Briefly, the reduction of AlamarBlue in the solution was determined per well by reading fluorescence at wavelengths of 545 nm excitation and 590 nm emission using a spectrophotometer (Molecular Devices iD3). A chondrogenic base medium, without extraction supplements, was used as a baseline to calculate % growth inhibition according to
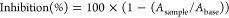
1where *A*_sample_ is the metabolic activity measured for each sample and *A*_base_ is the metabolic activity measured for cells cultured in a normal expansion medium. Cytotoxicity was determined as more than 30% cell growth inhibition, as per ISO 10993-5:2009.

### 2.8 Cell Attachment, Proliferation, and Morphology

Human bone marrow-derived stromal cells (hMSCs; ZenBio, Durham, USA) were expanded to passage 3 in the marrow stromal cells (MSCs) expansion medium [Gibco αMEM nucleosides glutaMAX supplemented with 10% (v/v) FBS, 100 U/mL penicillin, and 0.1 mg/mL streptomycin], incubated at 37 °C in a humidified air incubator (5% CO_2_/95% air). All scaffolds were cut into Ø6 mm samples, sterilized in 70% ethanol for 30 min, washed in PBS (3×), and placed in ultralow attachment plates (Corning Costar). Each scaffold was seeded with 100,000 cells and subsequently cultured in the osteogenic differentiation medium [Gibco αMEM nucleosides glutaMAX supplemented with 10% (v/v) FBS, 100 U/mL penicillin, and 0.1 mg/mL streptomycin, 10 nM dexamethasone, 10 mM Β-glycerophosphate, and 0.1 mM AsAp] for 3 weeks.

AlamarBlue was used to track cellular health and proliferation over time, following the manufacturer’s instructions. Samples were furthermore harvested at various time points and fixed in 4% formaldehyde for 1 h at RT, washed with PBS with 0.3 M glycine, and evaluated using immunohistochemistry techniques. In brief, samples were first incubated in PBS with 0.1 wt % Triton-X-100 for 6 min followed by 2 wt % BSA blocking buffer for 1 h at RT. Morphological evaluation was performed by staining for f-actin (20×, 1h) and DAPI (1000×, 20 min) in RT. Bone-specific extracellular matrix components were further stained using primary antibodies for collagen type I (450×) and osteopontin (25×) overnight at 4 °C. Samples were then washed in 2 wt % BSA before staining with secondary antibodies (GaM and DaR; 500×) for 1.5 h at RT followed by 20 min with DAPI and F-actin. All samples were imaged with an ECHO Revolve fluorescent microscope (BICO, Sweden).

## 3 Results

The initial step in assessing cryo-EHD jetting’s potential for high-resolution scaffold printing involved confirming its capability to generate a jet from a concentrated SF solution. We observed that by increasing the voltage above 3 kV and reducing the working distance below 3 mm, a continuous jet could be achieved independently of the collector temperature. This observation suggested sufficient molecular entanglement within the SF solutions, as demonstrated in Video S2. However, due to the flow properties of the SF aqueous solution (as illustrated in Figures 1b and S3a—rheology of 50% v/v SF solution), the jet loses its cylindrical shape upon contact with the collector.

**Figure 3:**
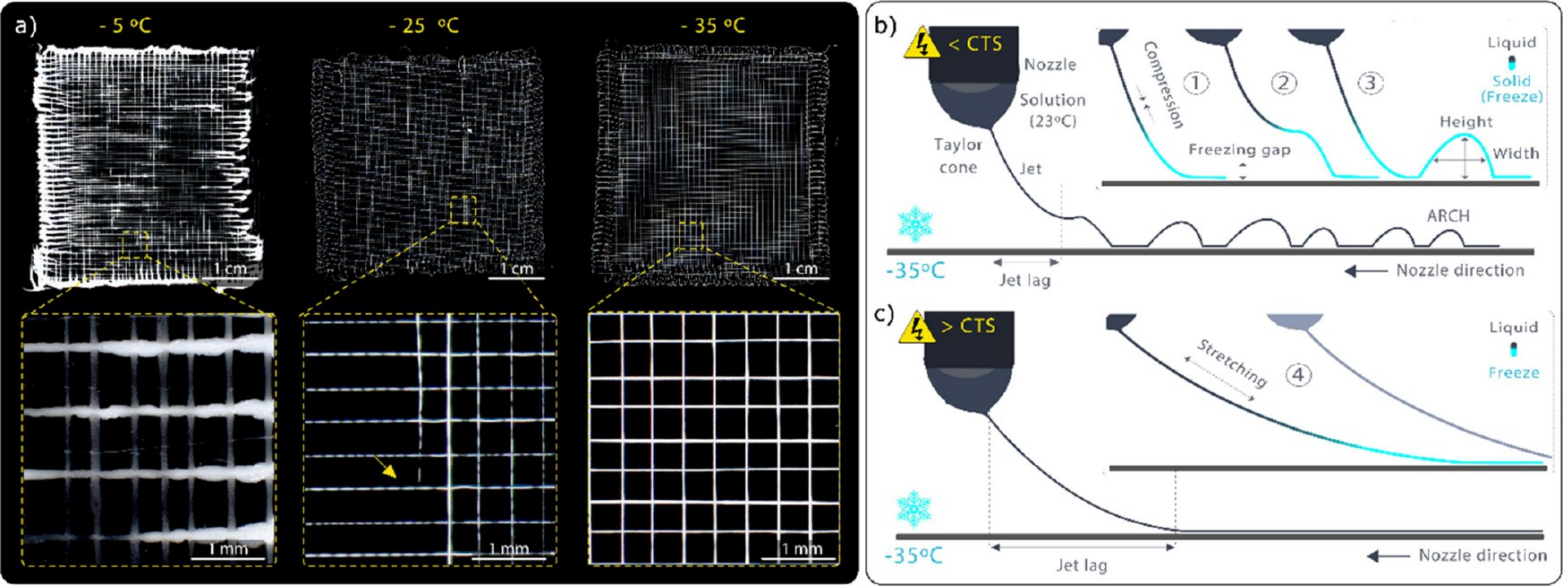
(a) Representative images of SF jet frozen at different collector temperatures. Stable jets are obtained when the temperature is set at −35 °C. Schematic representation of the behavior of fibers (b) below and (c) above the critical translation speed (CTS). Note that below CTS the jet forms arches due to the rapid fiber solidification and the compression forces that fast jet extrusion generates.

To determine the optimal solidification dynamics of the SF aqueous solution, the effect of the collector temperature was evaluated (Figure 3a). Repetitive samples were printed using a collector with a temperature that was gradually decreased, starting at −5 °C and reaching −35 °C in increments of −5 °C. The print model used was a square pattern with 30 mm of side, composed of two layers with parallel straight fibers spaced 500 μm apart and rotated 90° relative to each other (Figure S3b and G-code S1). This resulted in a scaffold made up of 3600 pores with square shape (60 × 60) of 0.25 mm^2^ each.

The printing accuracy and reproduction were substantially impacted by the collector temperature (as shown in Figure 3a). At a collector temperature above −10 °C, the jet did not instantly solidify upon contact with the cold surface, resulting in the formation of flattened “ribbons”. The jet was also not stable, leading to variations in fiber continuity, diameter, and volume. At −15 °C or below, the jet solidified upon contact with the collector, forming well-defined circular fibers. However, the jet was still displaying instabilities, causing fiber breakage (as indicated by the yellow arrow in Figure 3a at −25 °C), and making it difficult to produce a defect-free scaffold. We found that the highest printing stability was achieved when the collector was set to −35 °C, allowing for the production of high-quality scaffolds (as shown in Figure 3a for −35 °C and in Video S3).

To increase the control over SF deposition, the effect of collector speed was also investigated. Setting up the collector temperature to −35 °C, and solution to RT, straight lines were printed at varying speeds, from 4.2 to 50 mm·s^–1^. Surprisingly, the deposited jet did not coil at low collector speeds but instead formed vertical “arch-shaped” structures (Figure S3c,d).^35^ This peculiar arching effect has also been observed during MEW of polyvinylidene difluoride and nylon.^36,37^ In both previous cases, rapid solidification of the jet occurred, suggesting that the temperature difference (Δ*T*) between the printhead and the collector can be a critical factor in achieving quality fibers.

Furthermore, the dimensions of the arches are strongly affected by the collector speed, increasing in size as the printing speed decreases (Figure S3d). This suggests a correlation between the formation of arches and the compression forces exerted on the jet (Figure 3b,c).^35^ When the collector speed is greater than 25 mm·s^–1^, arches are not optically visible while the fibers are stretched and thinned out at 33.4 mm·min^–1^ (Figure S3d). A collector speed of 25 mm·s^–1^ was therefore chosen to prevent instabilities while achieving microfiber deposition. Compared to MEW with jet speeds of typically 6.3 mm·s^–1^ for PCL, the jet speed here is faster, perhaps due to the higher electrical conductivity and lower viscosity of SF solutions.

**Figure 4:**
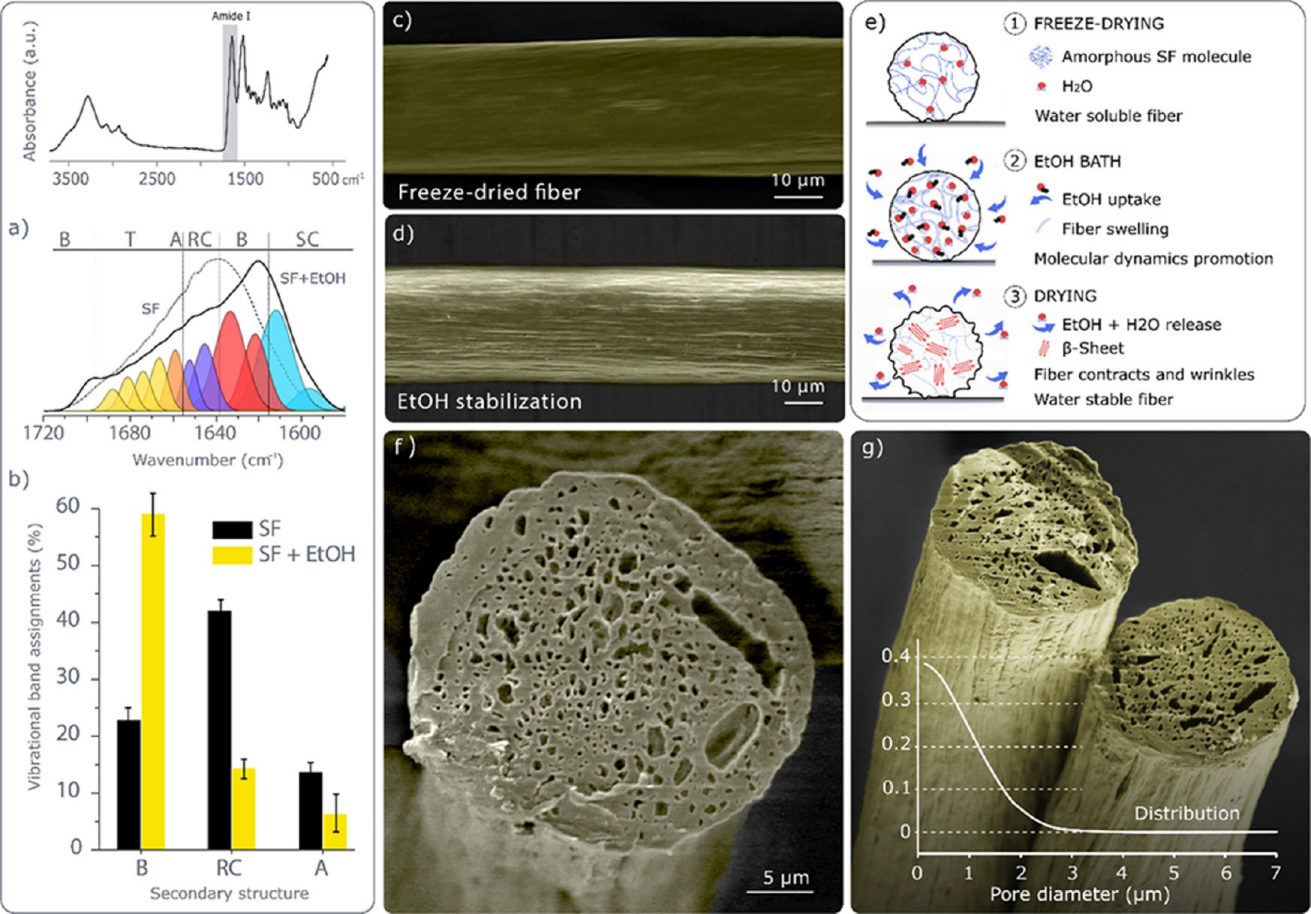
FTIR spectra of SF scaffolds after EtOH treatment: (a) Deconvolution of the amide I region for SF scaffolds before and after EtOH treatment—side chains (SC), β-sheets (B), random coil (RC), α-helix (A), and turns (T)-, and (b) vibrational band assignments for SF scaffolds before and after EtOH treatment. (c) SEM images of freeze-dried SF fiber before and (d) after an EtOH treatment. (e) Schematic of the ethanol treatment effect on SF fibers, (f) cross-section SEM image of freeze-dried SF fiber, and (g) distribution of pore diameter (fibers within SEM images are false-colored yellow).

The fibers, generated under stable processing conditions (listed in Table 1), were subsequently freeze-dried. These fibers display a cylindrical shape with a nonporous surface and an average diameter of 27 ± 5 μm. Additionally, they exhibit a notable degree of flexibility, as evidenced by manual twisting results, as depicted in Figure S3e,f. Analysis of the amide I peak from FTIR shows that the freeze-dried fibers are amorphous (Figure 4a), likely because of the jet's rapid freezing upon contact with the collector and the water molecules trapped inside the SF structure, which prevents molecular rearrangement and consequent SF crystallization. This results in water-soluble fibers (as demonstrated in Video S4). To prevent this solubility, the freeze-dried SF fibers were dipped in 80% ethanol (EtOH) and subsequently dried at RT, leading to water-stable scaffolds having a β-sheet content of approximately 60%^30^ (Figure 4b, and Video S4). The smooth surface of the fibers (Figure 4c) underwent some alterations during the EtOH dipping, exhibiting longitudinal wrinkles of 1.6 ± 0.5 μm wide and resembling a microfibrillar structure (Figure 4d). This shape change is believed to result from the approximate 50% diameter increase during EtOH treatment (Figure 4e).

The SEM images of the cross-section of water-stable fibers show a porous core and a solid outer layer (Figure 4f). The size distribution analysis shows that most of the pores have a diameter of less than 1 μm, although some have diameters up to 7 μm (Figure 4g). The longitudinal evaluation of the fibers shows that the pores are axially aligned and have a high aspect ratio, which can be observed with an optical microscope, where bubbles can be seen traveling within pores along the EtOH-swollen fibers (Video S5). Although it is difficult to determine, the shape of the fibers and pores seems to be related to the preferential nucleation and growth dynamics of ice crystals, the stretching of the fibers during printing, the drying process, and the high SF concentration.

To investigate alternative drying methods, specifically the “dry-defrost” approach, the just printed samples were positioned on a −20 °C aluminum block within a chamber exposed to a dry air stream. This method allowed for the gradual melting of ice crystals and the evaporation of the released water, resulting in efficient water removal, compact core structures, and the formation of optically transparent fibers (Figure S4a,b). However, it is important to note that for the majority of experiments, we opted to use freeze-dried samples treated with ethanol stabilization as the default approach.

**Figure 5:**
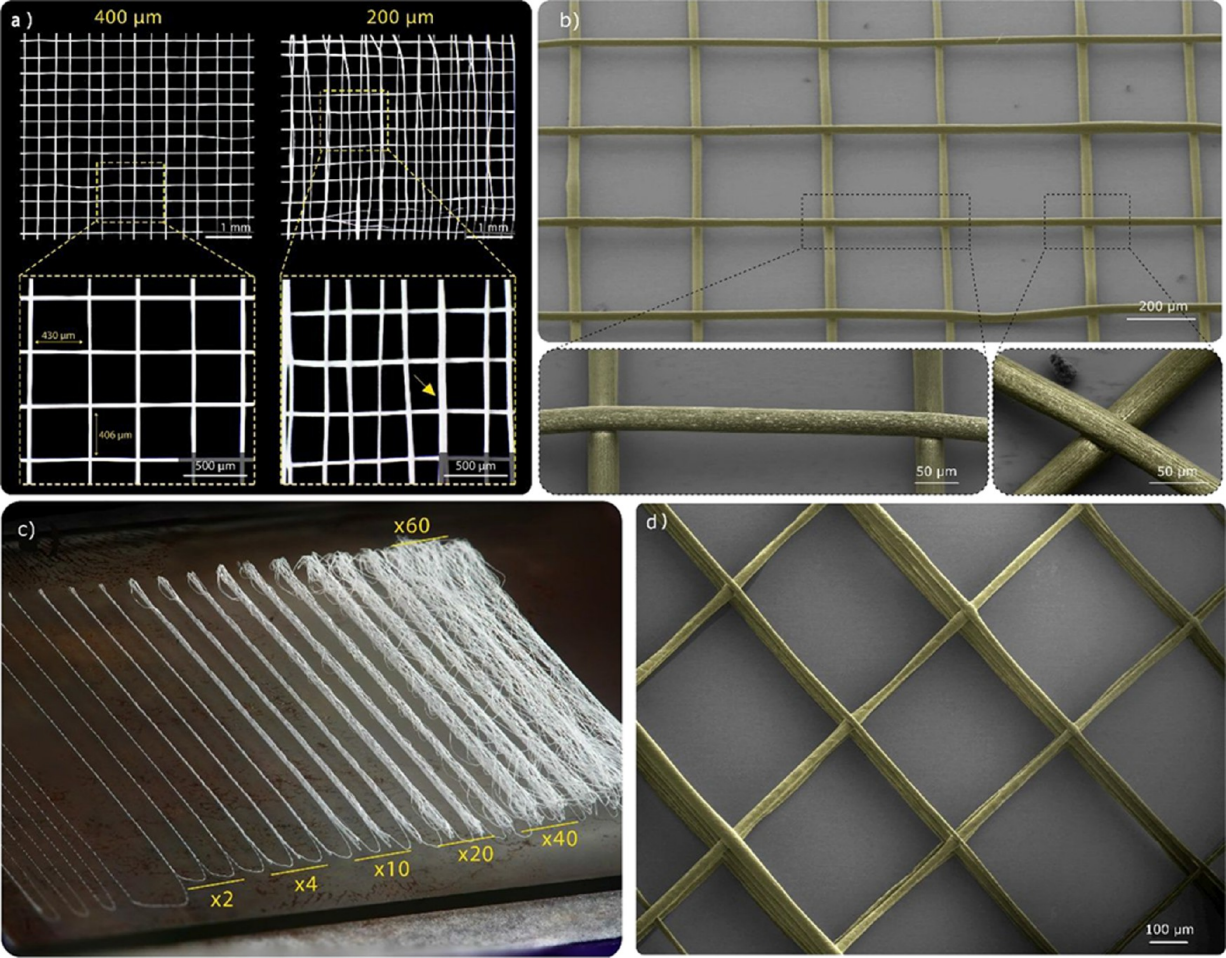
Representative images of SF scaffolds: (a) Dark-field stereomicroscope images showcasing square SF structures featuring a single layer with variable fiber interspace (400 and 200 μm). Notably, electrostatic forces lead to fiber repulsion or attraction when the interspace is 200 μm or smaller. (b) Top: scanning electron microscope (SEM) image reveals square SF structures with a single layer and a 400 μm interspace. Bottom: SEM image offering a detailed view of SF fiber bridging (left) and fiber adhesion (right). (c) Depiction of stacked SF fibers forming walls of varying heights. These fibers rapidly freeze upon contact with the collector or adjacent fibers. (d) SEM image depicting square SF structures with a 400 μm interspace and 12 stacked layers. The precision of the printing process allows for the construction of perfectly vertical walls. All fibers within SEM images are false-colored yellow.

To investigate the accuracy limits of SF jetting, scaffolds with different fiber interspacing were designed by using G-codes S1, S2, S3, and S4. These G-codes define square scaffolds of 30 mm edge length, two stacked layers rotated 90° with respect to each other, and parallel lines with varying interspacing (500, 400, 300, and 200 μm, respectively) (as shown in Figure S4c). Fiber deposition accuracy was achieved when the fiber interspacing was 400 μm or greater (Figures 5a and S4d). Below 400 μm spacing, however, fiber deposition accuracy was reduced, likely due to charge accumulation and the electrostatic repulsion/attraction forces acting on the jet and deposited fiber.^38−40^ Additionally, it was observed that most of the SF fibers in the second layer of the scaffold were overhanging (bridged) (as shown in Figure 5b). This suggests that the fibers solidified quickly, even when not in direct contact with the frozen collector, which opens the possibility of further increasing the height of the scaffold by multiple layers stacking.^39^ In all cases, the stacked fibers were fused together, ensuring adhesion between layers.

The potential of cryo-EHD jetting using aqueous solutions was further assessed by creating fiber arrays of varying heights (Figure 5c). Arrays consisting of two stacked layers of up to 60 layers were designed (G-code S5 and Figure S4e). Regardless of the array’s size, the fibers solidified upon contact with the collector or when they came into contact with previously deposited fibers. Notably, the fibers retained their well-defined fibrillar shape even when the distance from the cooling source was increased, confirming the cryo-EHD jetting’s capability to build vertically. Deposition accuracy remained consistent up to 20 layers, but beyond that, the accumulation of charge led to fiber repulsion and a loss of deposition control^39^ (Figure 5c). It must be considered that the printing speed and straightforward array patterning result in a limited time for fiber discharge. This leads to the accumulation of charge, which amplifies the attraction/repulsion effect among fibers, resulting in a loss of deposition accuracy after a few layers. This effect can be mitigated by increasing the time it takes for the jets to settle between layers.

Scaffolds with multiple layers were printed using a 0/90° square laydown pattern of 500 μm spacing and 30 mm size with a total of 12 layers (Figures 5d, S4f and G-Code S6). Those samples were produced in approximately 7 min, resulting in a stable and continuous jet of around 30 m of SF fiber (Video S3). This speed of fabrication is notably faster than conventional MEW of PCL, which typically uses collector speeds between 6 and 7 mm·s^–1^, requiring around six times more time to produce the same structure.^41^ Despite some printing failures and occasional defects, the SF scaffolds displayed well-defined pores, homogeneous fiber interspacing, good stacking, and consistent fiber homogeneity.

As a final experiment to assess the alternative drying process, we investigated the combination of dry-defrosting with freeze-drying to create a scaffold with varying porosity within individual layers. To achieve this, freshly jetted scaffolds were first placed on a −20 °C aluminum plate exposed to a dry airflow for 30 min. Subsequently, they underwent a freeze-drying process. The resulting scaffolds displayed a heterogeneous structure, with a gradual reduction in porosity as the fibers moved farther away from the aluminum collector plate, where they remained frozen for an extended duration (Figure S4g,h).

It is important to emphasize that regardless of the drying process and whether a stabilization process was applied, the secure attachment between layers ensured the stability and manageability of the scaffolds (Figure S5). It was observed that, in some instances, the stresses during the drying process led to warping of the shape of the scaffolds.

**Figure 6:**
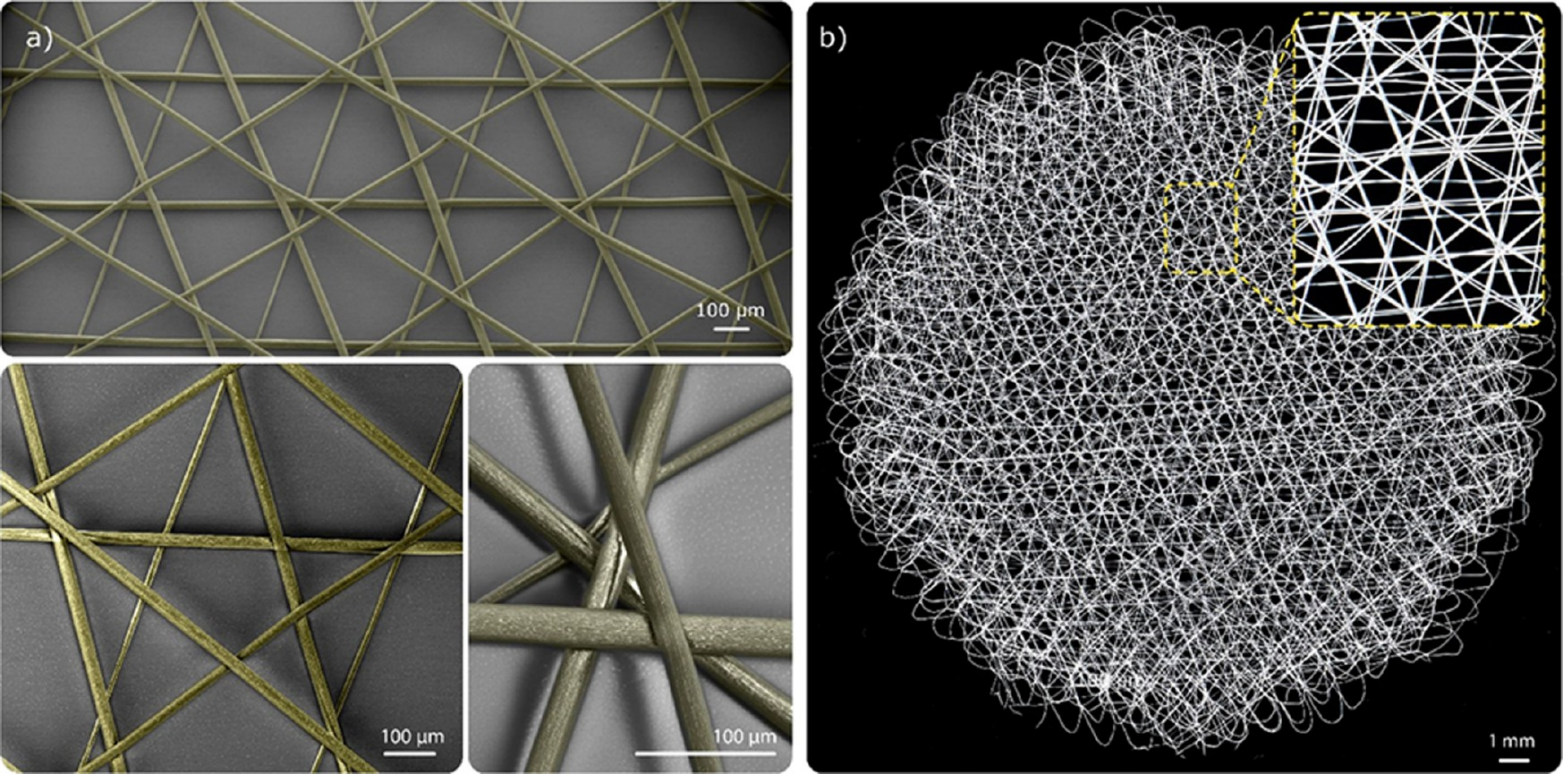
(a) SEM images of a hexagonal shaped SF scaffold with one layer. The obtained structures match the predesigned patterns. (b) Microscope image of same scaffold with 25 layers. All fibers within SEM images are false-colored yellow.

Finally, the exploration of more complex geometries was conducted. For this purpose, first, a 25 mm diameter hexagon scaffold with straight fiber patterns of 500 μm fiber interspace and 72° rotated layers was designed and printed using G-code S7. The resulting scaffold had patterns and pores that matched the toolpath (Figures 6a versus S6a), highlighting the control of fiber deposition and its suitability for printing both micro- and macro-structures. Additionally, scaffolds with multiple repeating layers were printed (G-code S8), where a stable jet could be observed for up to 25 stacked layers (Figures 6b and S6b).

As an additional test, the ability to print sinusoidal-shaped fibers was investigated (G-code S9). Fibers with controllable coils and amplitude were obtained, with the amplitude of achieved sinusoids less pronounced than the toolpath, due to the printing speed and corresponding jet lag (Figure S7).

**Figure 7:**
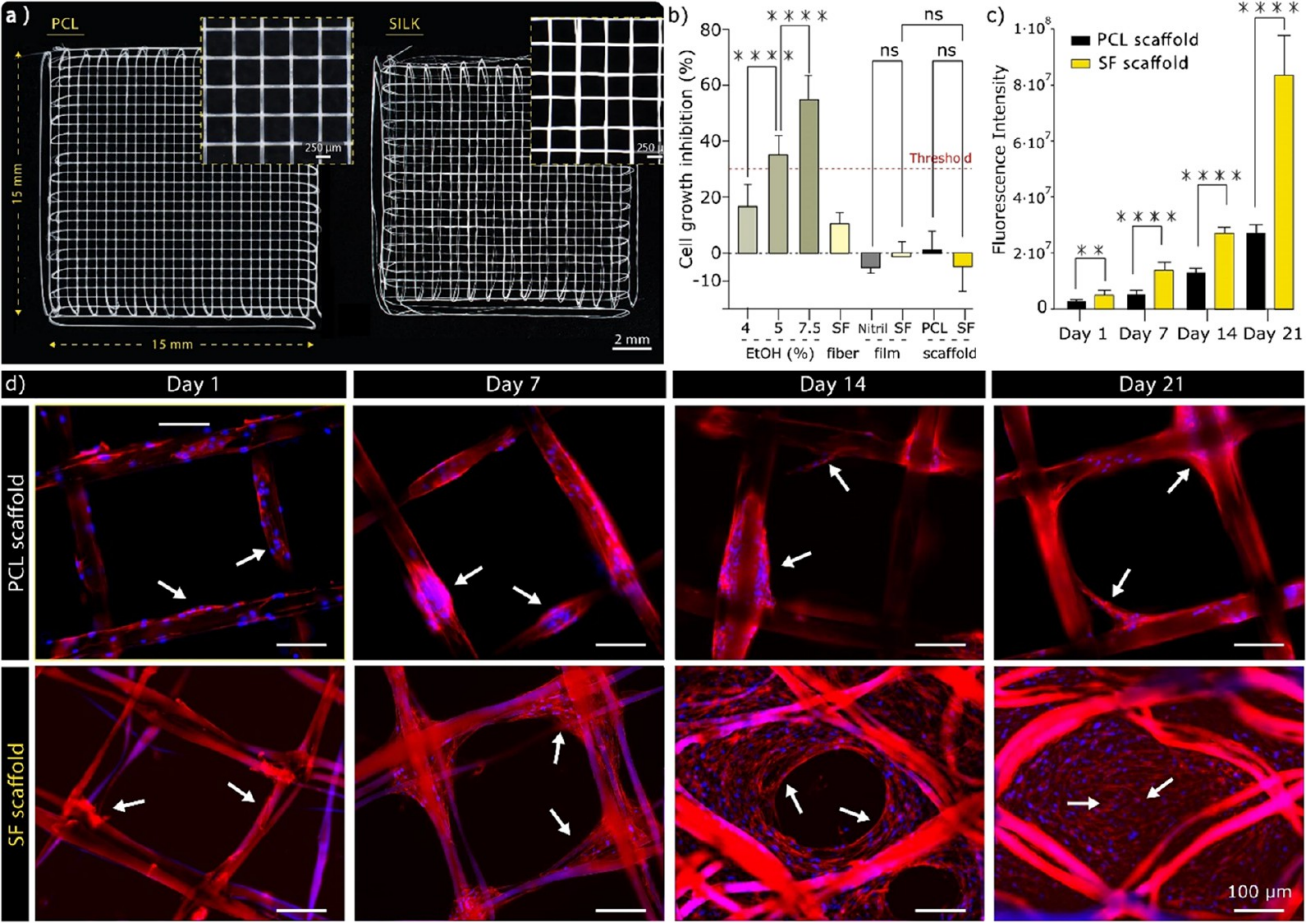
(a) Representative images of PCL and SF scaffolds of 15 mm edge length, 500 μm fiber spacing, and 10 layers printed for biological assays. (b) Growth of human dermal fibroblasts (hDF), measured with AlamarBlue, after exposure to concentration gradients of ethanol. Cell growth inhibition is calculated relative to the media baseline. Red line indicates the 30% cell growth inhibition threshold for cytotoxic responses. Error bars represent the mean ± SD of nine samples (three repeats). **** indicates a significant difference of *p* < 0.0001 while ns denotes a nonsignificant difference of *p* > 0.05. (c) Metabolic activity/cell growth of human dermal fibroblasts (hDF) as a function of time, measured with the AlamarBlue assay. Error bars represent the mean ± standard deviation of nine samples (three repeats). **** Indicates a significant difference of *p* < 0.000001 while ** denotes a significant difference of *p* < 0.01. (d) Visualization of bone marrow-derived stromal cell (hMSC) morphology as a function of time and scaffold type. Molecular probes DAPI for cell nuclei (blue), and rhodamine-phalloidin for F-actin (red). The cells are mostly located as cellular clusters along the PCL scaffold junctions while cells on silk fibers are growing into the scaffold pores forming a confluent tissue over the culture period, as indicated by white arrows. Scale bar = 100 μm.

To ensure that the basic biocompatibility remains after these processing steps, fundamental in vitro analyses of the SF scaffolds were performed on 15 mm side square scaffolds with a 500 μm fiber interspace and 10 layers (Figure 7a). PCL scaffolds were used as controls since it is the most common polymer used for MEW. In an effort to provide a comparative study relevant to the MEW community, alongside SF scaffolds, similar-sized structures fabricated using traditional MEW and medical-grade PCL were developed and compared in vitro.

The biological compatibility of the SF material and the EHD process was evaluated using an in vitro cytotoxicity testing assay in accordance with ISO10993-5. In this regard, extracts of degummed SF fibers, SF films, and EHD processed SF scaffolds were tested for cell growth inhibition using hDFs. In addition to PCL scaffold controls, extracts of synthetic rubber (nitrile) films were included as reference materials for the ISO10993-5 protocol together with serial dilutions of ethanol as positive cell growth inhibition controls to validate the assay. Results revealed that degummed silk fibers yielded an 11.6% inhibition of cell growth (Figure 7b). Although these findings do not fall under the category of cytotoxicity (with less than 30% inhibition), they suggest that the degummed silk fibers sourced in this study may release residue compounds that influence cellular growth. These residues likely originate from the degumming process and may be associated with traces of Na_2_CO_3_ on the fibers. Interestingly, both silk films and printed silk scaffolds were observed to be highly cytocompatible, similar to both the nitrile and PCL materials utilized as negative controls (Figure 7b). This reflects the successful removal of any cytotoxic residues during the SF material preparation prior to the EHD jetting, which includes both solubilizing the silk in a LiBr aqueous solution and dialyzing it prior to downstream fabrication.

To further validate the ability of EDH jetted silk structures to be used as scaffolds for downstream tissue engineering applications, another systematic study was conducted to determine cell attachment and proliferation. To this end, SF scaffolds were seeded with human bone marrow-derived stromal cells (hMSCs), a clinically relevant cell source that can be used for a wide range of tissue engineering applications. PCL scaffolds were again used as controls to provide meaningful interpretations. It was confirmed herein that SF scaffolds supported cellular attachment and proliferation of MSCs. Specifically, a 32-fold increase in metabolic activity was observed for cells seeded in SF scaffolds over the culture period (Figure 7c). The control scaffolds (PCL) also confirmed successful cell attachment and a significant proliferation, with a ninefold increase in metabolic activity following 3 weeks of culture (Figure 7c). Cells seeded on either type of substrate (PCL or silk) were able to maintain an elongated morphology throughout the culture period, with f-acting stretching both directly along the 3D-printed fibers and along cluster of cells (Figure 7d). Owing to the rapid proliferation of MSCs in SF scaffolds, the cells were able to also grow into the scaffold’s pores forming a confluent matrix after 3 weeks of culture (white arrows, Figure 7d). Cells seeded onto PCL scaffolds were instead observed to grow into highly dense cell clusters, accumulating around the fiber junctions of the scaffolds (white arrows, Figure 7d). This underscores the long-standing dilemma with achieving enhanced printing resolution and process control through the use of synthetic materials and the inherent lack of cell–material interactions. The ability to herein remove these traditional design restrictions through EDH processing of silk into scaffolds with both high printing resolution and maintained bioactivity opens up for expansive applications of EDH jetting across both 3D-printing and tissue engineering fields.

## 4 Discussion

This study primarily focuses on addressing the processing challenge of controlling the EHD jetting of SF solutions. This involved reducing system instabilities, regulating the rapid deposition of jets and facilitating the swift solidification of the ejected material. Previous efforts to achieve rapid solidification using UV-curable systems and cryogenic baths have been successful for processing aqueous solutions but have frequently led to limited jet stability and deposition accuracy. Additionally, many of these approaches required the use of additives to modify solution rheological properties or cross-linking dynamics. Overcoming these challenges was pivotal in advancing the technology to a more mature stage and facilitating the transition from laboratory-scale experimentation to a more productive system. In this study, the processed solution is composed of only SF protein and water, and organic solvents and additives are avoided. This represents a first step toward process sustainability, from where further studies can use SF scaffolds in various applications.

The continuous and stable jetting achieved here allows for the precise deposition of centimeter-scale scaffolds in a rapid, repeatable manner. This remarkable jet stability can be attributed to the delicate balance between molecular entanglement and jet elasticity, influenced by the jet’s temperature.^31^ Increasing the SF concentration up to 50% v/v allows us to obtain a highly viscous solution. The molecular weight of SF chains, with the large solute concentration, increased molecular entanglements, which are essential for pulling the solution once the jet has been formed. The molecular entanglements, along with a reduction in molecular dynamics as the jet approaches the collector, significantly enhance jet elasticity.

One of the primary challenges when printing at low temperatures is the risk of water condensation on the cold surface and the subsequent growth of ice crystals. Excessive ice crystal growth can obstruct the collector and lead to printing instabilities, ultimately preventing continuous jet printing. The experiments conducted at −35 °C demonstrated negligible ice crystal growth during the extended printing of scaffolds, confirming the effectiveness of maintaining a dry environment to prevent ice crystal growth during printing. It is worth noting the dual gas blowing system, which directs dry air both into the chamber and directly onto the collector surface. By implementing this system, we were able to control whether dry air was directly applied to the collector surface. While there were no significant differences observed in jet stability when air was directly blown onto the surface, a slight improvement in layer stacking was achieved. The slight improvement in layer stacking can be attributed to the removal of surface charge due to accelerated water evaporation, leading to reduced charge accumulation and a subsequent decrease in interfiber attraction/repulsion forces. However, it is necessary to further study the charge accumulation in highly concentrated solutions to achieve better control over the jet stability and deposition.

The solution’s higher conductivity and lower viscosity compared to molten polymers led to a larger solution attraction when applying an electrical field, resulting in faster jets. Moreover, the open-source cryo-EHD jetting device allowed us to move the printer head at speeds as fast as 42 mm·s^–1^, improving scaffold production.

Another noteworthy observation was the “arching effect” of fibers when jets were deposited onto the frozen collector at slow collector speeds. Our hypothesis is that the arches are a consequence of premature freezing of the jet before contact with the collector (as depicted in Figure 3b). The process initiates due to jet lag and instabilities, causing the fiber to start solidifying perpendicular to the printing plane, reducing the fiber’s coiling freedom and inducing it to overhang. Subsequently, the solidification of the fiber progresses, increasing the length and height of the overhangs until the fiber moves far enough away from the frozen collector. At this point, the jet stops freezing sufficiently fast, loses stiffness, and falls back to the collector. Therefore, it can be deduced that, for a constant jet speed, the smaller the printing speed, the greater the compression forces exerted on the jet.^3^ As a result, the arch dimensions are inversely proportional to the printing speed.

The printed scaffolds were successfully stored in a freezer until the drying process. The primary drying process, i.e., freeze-drying, resulted in fibers with micrometer and elongated pores, potentially expanding the applicability of EHD structures in various fields. The alternative dry-defrost process provided a simple strategy to obtain compact fibers and slightly reduce their dimensions compared to those of freeze-dried ones. In all cases, the use of water as a solvent facilitated the process and eliminated the need for complex ventilation and drying systems.

Further, the choice of water as a solvent paved the way for implementing printed structures as scaffolds to support cell attachment and proliferation. Traditionally, thermoplastic polymers have predominantly been used for high-resolution EHD structure printing, often possessing limited bifunctionality. The processing of high-resolution SF structures, along with their promising cellular response, opens up new avenues for tissue engineering.

Regarding the developed cryo-EHD jetting system, all components were designed to be swappable and modular, with the intention of using the Voron hardware as a platform for assembling different components to enable various printing modes. This approach can be integrated into the MEWron project, which aims to improve the accessibility of the MEW devices. In this specific case, the simplicity of the printhead made of 3D-printed plastic parts enabled easy electrical isolation of all components. The collector, which contained a Peltier device with two ceramic surfaces, also facilitated this work. As a result, we obtained a simple and easily reproducible high-quality device with submicrometric accuracy.^11^

Future system updates may address the need for a multicomponent solution, integrating all of the required components into a single unit. This task is made feasible by the integrated Raspberry Pi, which acts as the brain of the printer and can efficiently manage multiple devices simultaneously. Notably, the Raspberry Pi can directly supply the required current for Peltier control, seamlessly integrate an open-source syringe pump into the printhead,^42^ and efficiently control an open-source high-voltage system.^43^

The final challenge revolves around improving the dissipation of heat generated by the Peltier device. This objective can potentially be achieved by utilizing predesigned central processing unit (CPU) and graphics processing unit (GPU) cooling systems, particularly all-in-one liquid refrigeration systems known for their excellent heat removal capabilities, all within a budget-friendly cost range. Furthermore, these cooling systems can also be conveniently controlled by Raspberry Pi.

## 5 Conclusions

In this study, we have demonstrated a viable process for aqueous solution EHD jetting, employing sustainable, straightforward, and low-toxicity methodologies. The use of a frozen collector at −35 °C has proven to be a simple and effective approach for producing homogeneous and stable fibers, which can be precisely stacked to create high-resolution 3D scaffolds. The viscosity and molecular entanglement of the solution have emerged as critical factors in reducing printing instabilities and ensuring stable prints, resulting in the formation of unique porous fibrillar structures. Additionally, the drying process can be harnessed to modify and control fiber properties, including the production of anisotropic scaffolds with varying or progressive properties, along different axes.

This work not only demonstrates a promising avenue for processing new water-soluble materials using EHD jetting but also underscores the need for alternative cross-linking and solidification methods to ensure water stability in the case of other polymers. Such methods could involve UV or visible light cross-linking, pH adjustments, or the use of vapors for chemical cross-linking while the fibers are in their frozen or dehydrated state. The deposition and stacking accuracy achieved with SF fibers represent a significant step in bridging the gap between MEW and aqueous solution EHD printing, although further research is warranted to enhance the process fidelity. Moreover, the high collector speed employed in this study has reduced scaffold production time, a notable advantage for scaling up production in future applications.

In conclusion, the research conducted in this study was made possible through the modification of an open-source 3D printer, offering a cost-effective and adaptable approach tailored to specific research objectives. The ability to control both macroshape and local microfeatures of the scaffolds highlights the immense potential of EHD technologies in transforming materials into novel forms and presents exciting opportunities for future research endeavors. This is particularly significant in the context of aqueous solutions, which encompass biologically derived polymers traditionally deemed incompatible with melt processing techniques. This study thus opens new horizons for materials processing and advances our understanding of EHD technologies, especially in the realm of biocompatible materials.

## Supporting Information Available

Extended data about the Cryo-electrohydrodynamic Jetting device and the results obtained from silk fibroin aqueous solution processing; Figure S1 offers an overview of the cryo-electrohydrodynamic (EHD) jetting experimental setup, including an infrared (IR) image emphasizing the collector’s temperature insulation, a graph depicting the time-dependent decline in relative humidity with the introduction of dry air, and an image showcasing the primary devices involved in the cryo-EHD jetting system’s operation; Figure S2 provides an in-depth view of the modifications performed on the Voron 0.1 3D printer to convert it into a cryo-EHD jetting device; Figure S3 shows the rheological properties of a 50% v/v SF solution, graphical representation of G-code S1 for scaffold creation, varied SF fiber production at different speeds, and SEM images of freeze-dried SF microfibers manipulated into spiral and knot shapes; Figure S4 highlights SF fiber cross-sections after an alternative drying process called dry defrost, graphical representations of G-codes for scaffold designs with varying pore sizes and interfiber spacing, images of square scaffolds, and close-up views of freeze-dried square scaffolds printed following different G-codes; Figure S5 provides a microscopic overview of SF printed scaffolds; Figure S6 shows a graphic representation and optical images of complex scaffolds, while Figure S7 presents a comparison between printed sinusoidal fibers and the originally designed toolpath (PDF)
G-code (ZIP)
Cryo-electrohydrodynamic Jetting device 3D models (ZIP)
Video S1: Voron 0.1 conversion into EHD 3D printer with a frozen collector, including an overview of new used components, and different elements working mechanism; Video S2: macroscopic view of jet stability during EHD printing on frozen collector; Video S3: continuous shot of defect-free scaffold printing into the frozen collector for 7 min; Video S4: SF scaffolds water stability before and after EtOH treatment; and Video S5: air bubble traveling along SF microfibers (ZIP)
