# Supplementary material for: Cryo-Electrohydrodynamic Jetting of Aqueous Silk Fibroin Solutions
Source: ACS Biomater Sci Eng. 2023 Nov 21;10(3):1843–55. doi: 10.1021/acsbiomaterials.3c00851 (PMC10934238; doi:10.1021/acsbiomaterials.3c00851)
Supplement: Supplementary file 1 — ab3c00851_si_001.pdf [file ab3c00851_si_001.pdf]

Cryo-electrohydrodynamic Jetting of Aqueous Silk Fibroin Solutions

Ander Reizabal[1,2,*], Paula G. Saiz[1,3,*], Simon Luposchainsky[1], Ievgenii Liashenko[1], DeShea Chasko[1], Senentxu Lanceros-Méndez[4], Gabriella Lindberg[1], Paul D. Dalton[1*]

1) Phil and Penny Knight Campus for Accelerating Scientific Impact,
University of Oregon, 1505 Franklin Boulevard, Eugene 97403, OR, USA
E-mail: ander.reizabal@bcmaterials.net and pdalton@uoregon.edu

2) BCMaterials, Basque Center for Materials, Applications and Nanostructures,
Bldg. Martina Casiano, UPV/EHU Science Park, Barrio Sarriena s/n, 48940 Leioa, Spain

3) Macromolecular Chemistry Group (LABQUIMAC), Department of Physical Chemistry, Faculty of Science and Technology, University of the Basque Country (UPV/EHU),
Barrio Sarriena s/n, E-48940, Leioa, Spain

4) Ikerbasque, Basque Foundation for Science, Bilbao 48009, Spain

Keywords: Additive manufacturing, near-field electrospinning, cryogenic, electrowriting, biomaterials.

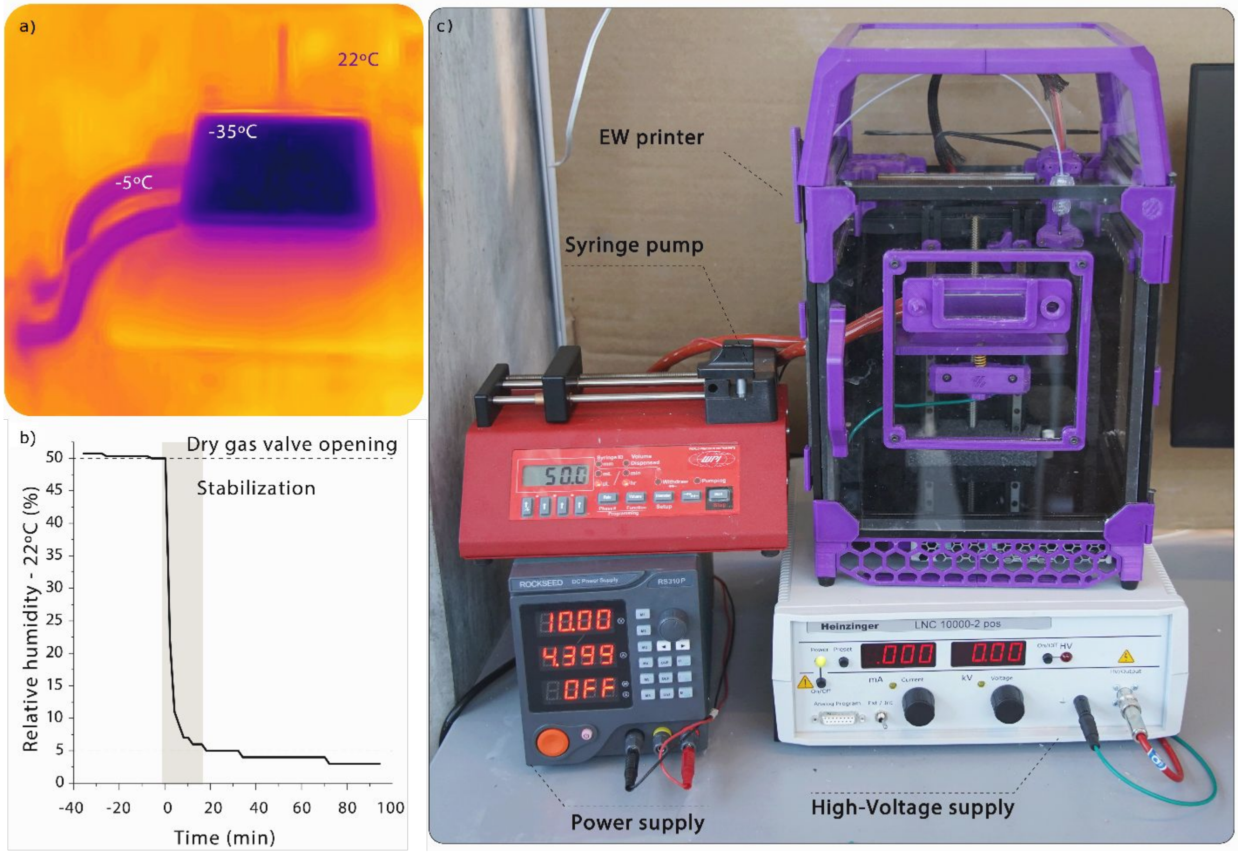

Figure S1. *a) This infrared (IR) image displays the cryo-EHD jetting device and emphasizes the collector's temperature. The 3D-printed plastic components of the collector effectively insulate and maintain low temperatures. The Peltier device generated heat, induces minimal temperature variation into the water/glycerol solution before and after coming into contact with the hot surface. b) The graph illustrates the time-dependent decrease in relative humidity due to the introduction of dry air inside the cryo-EHD jetting device enclosure. A sharp decline in humidity is observed as soon as the dry air begins to circulate within the chamber. Relative humidity levels drop below 5% within 20 minutes, reaching values around 2% after 60-80 minutes of stabilization. c) This image provides an overview of the cryo-EHD jetting experimental setup, showcasing the primary devices involved in its operation: a syringe pump for flow rate control, a DC power supply to regulate the cooling capacity of the Peltier device, a high-power DC supply to generate the necessary electrical fields for EHD printing, and the cryo-EHD jetting system itself.*

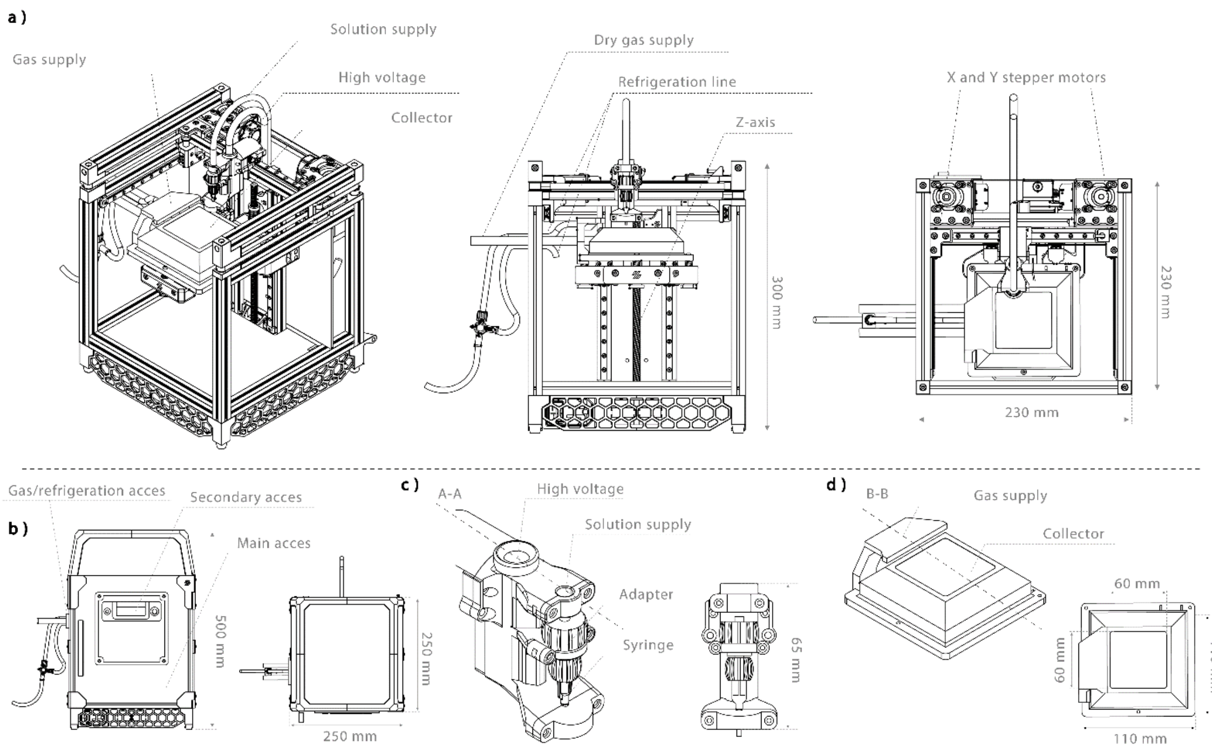

Figure S2. *a) This section provides an in-depth look at the modifications made to the Voron 0.1 printer to transform it into a cryo-EHD jetting device. Three primary modifications were carried out. b) An enclosure was added to regulate the environmental conditions and prevent the condensation of gases onto the collector. Additionally, a secondary access point was incorporated into the main enclosure door to simplify the replacement of samples. c) The original printhead was replaced with a 3D-printed alternative capable of accommodating nozzles of varying sizes. This new printhead connects to a syringe pump responsible for regulating flow rates and a high-voltage power supply required for the printing process. d) The freeze collector, temperature-controlled by a Peltier thermoelectric device, can lower the surface temperature to as low as -45°C. It features an additional gas supply system that delivers dry air directly to the collector's surface.*

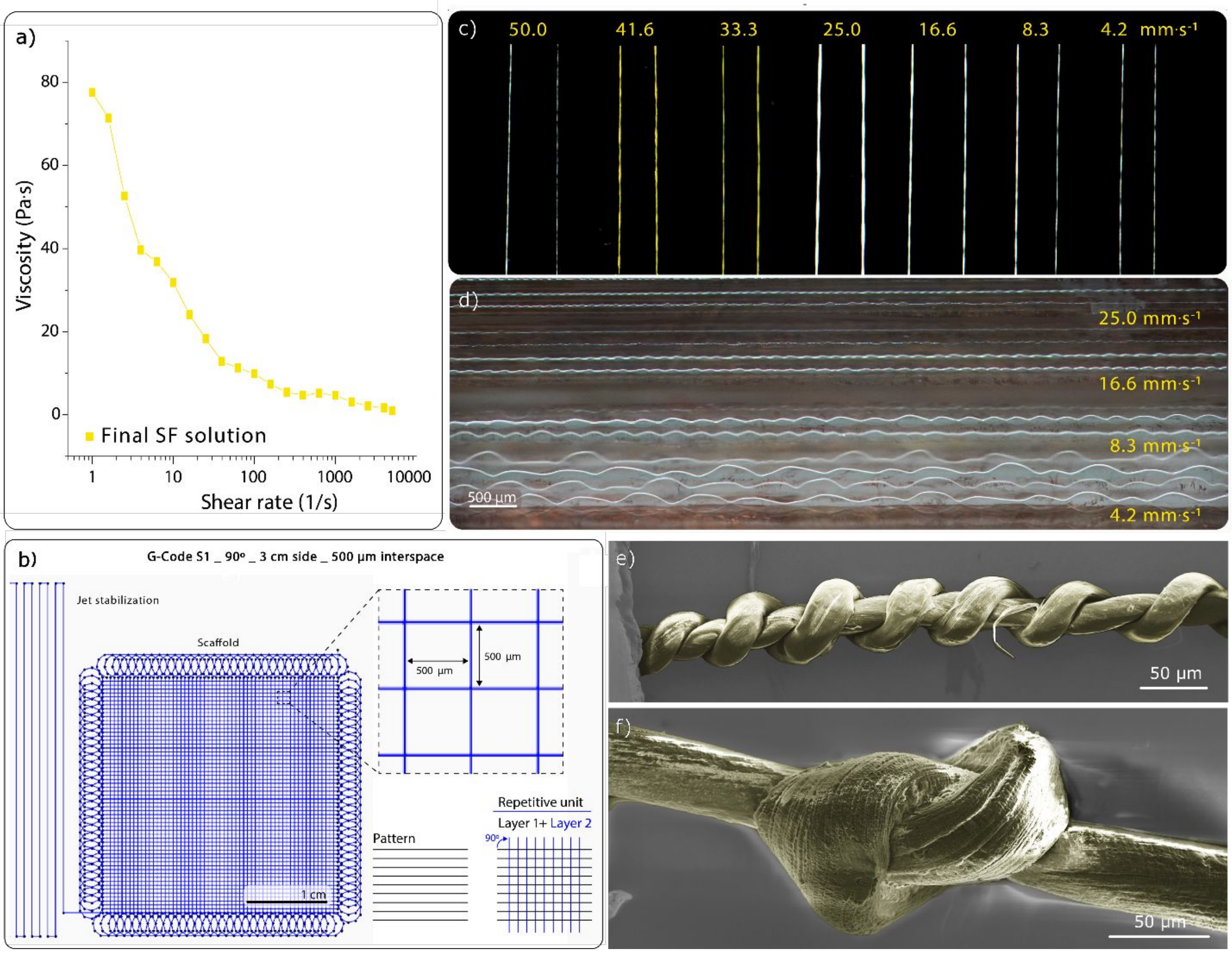

Figure S3. *a) 50% v/v SF solution rheology under variable shear rates. b) G-Code S1 graphical representation, fiber spacing, and layer rotation. The intersection of two adjacent fiber arrays resulted in a scaffold made up of 3,600 pores with square shape (60 x 60) of 0.25 mm² each. SF fibers produced at different printing speeds, c) top and d) side views. SEM images of freeze-dried SF microfibers after cryo-EHD jetting twisted manually to form a a) spiral and b) a knot.*

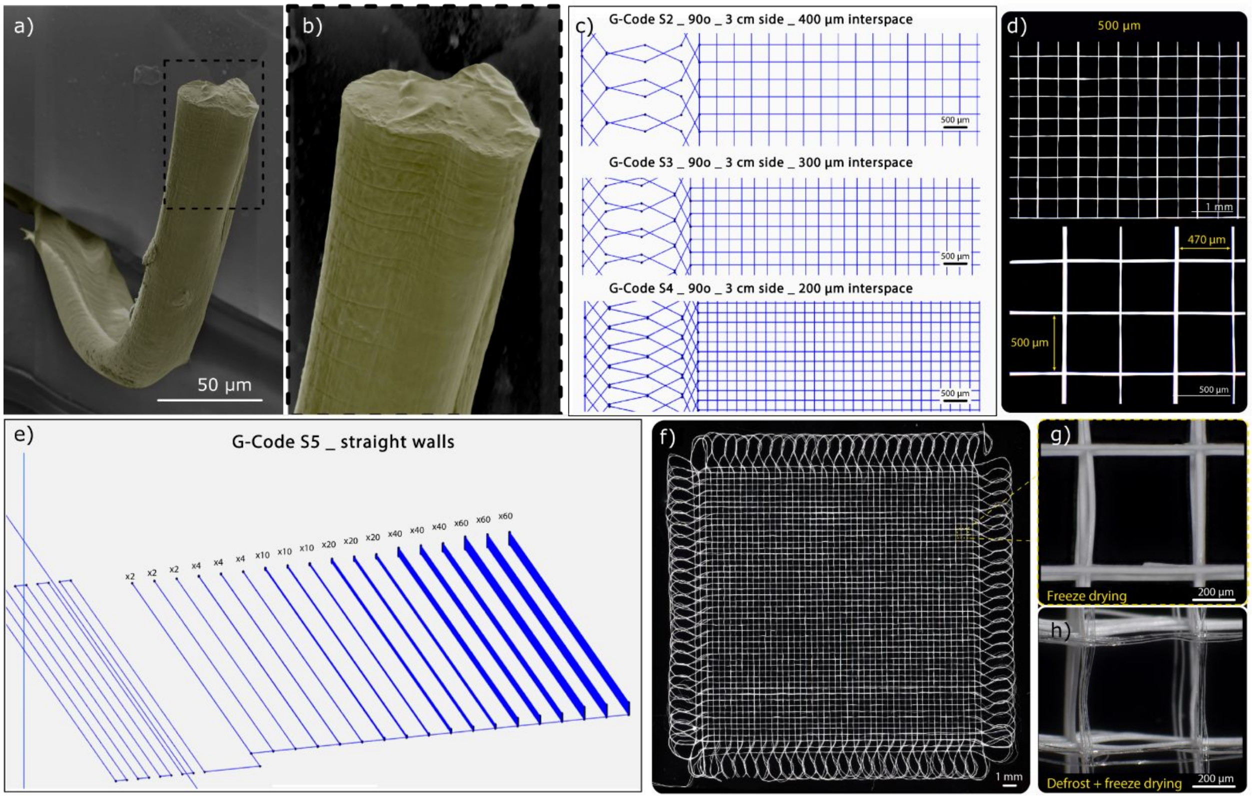

Figure S4. *a) SEM images of SF fiber cross-sections after an alternative drying process (dry defrost). The gradual removal of water during fiber defrosting allows for the elimination of trapped water, resulting in compact fibers. b) Zoomed-in view of the cross-section of fibers subjected to dry defrosting. c) Graphical representation of G-Code S2 to S4. These codes define square scaffolds with varying interfiber spacing and pore sizes, ranging from 400 µm to 200 µm. d) Dark-field stereomicroscope images depicting scaffolds with 500 µm interfiber spacing, obtained from G-Code S1. The final spacing between fibers is slightly reduced from the designed G-Codes due to variations in fiber diameter. e) Graphical representation of G-Code S5, illustrating walls with varying numbers of stacked layers, starting at 2 layers and reaching a maximum of 60 layers. f) A 40 mm side square scaffold with a 500 µm pore size, consisting of 12 layers (G-Code S7). Scaffolds were produced continuously, resulting in a stable and continuous jet of around 30 meters of SF fiber. g) Close-up view of the freeze-dried square scaffold printed following G-Code S7. h) Close-up view of a square scaffold initially subjected to dry defrosting and subsequently freeze-dried.*

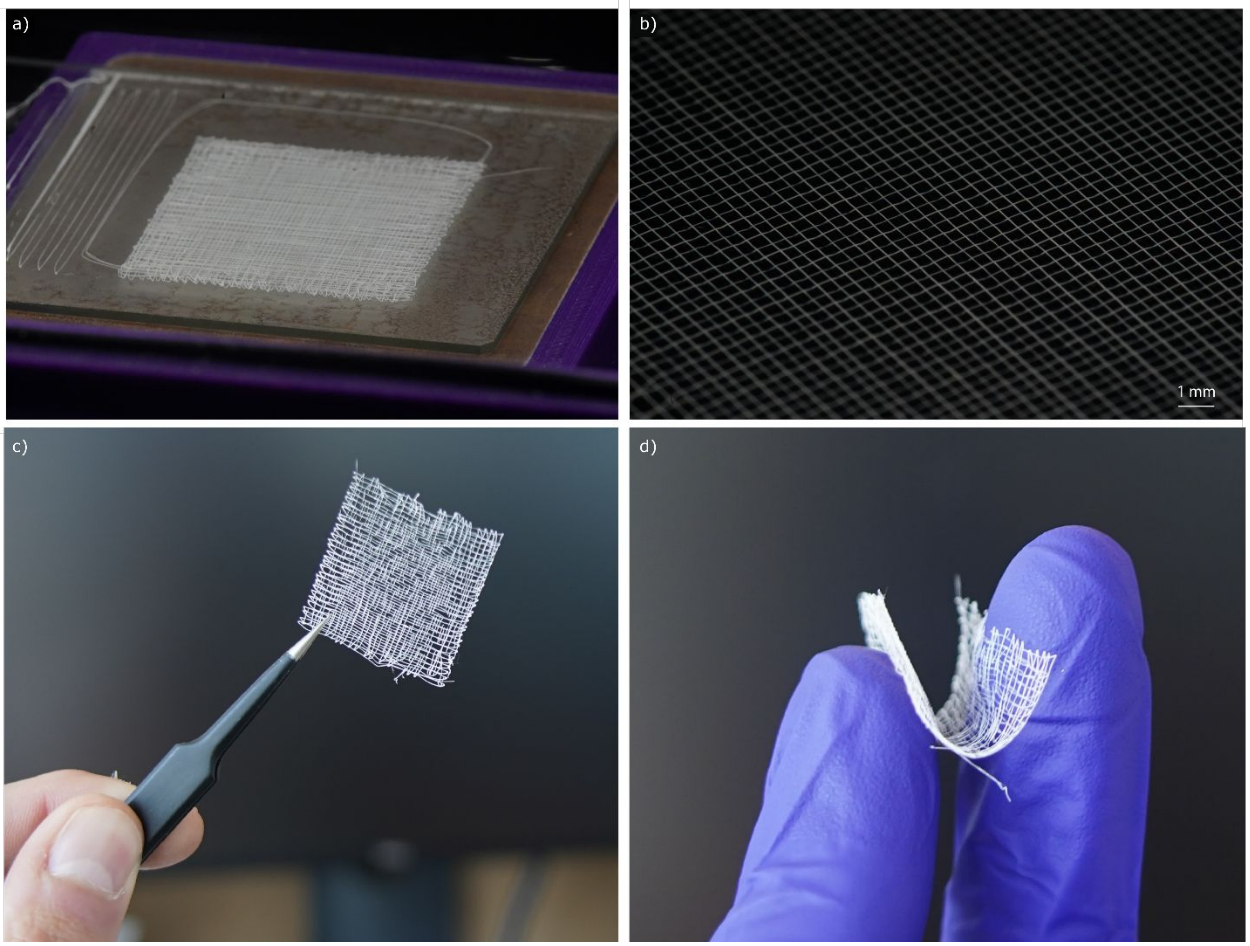

Figure S5. *a) SF scaffold before freeze drying. b) freeze dried square scaffold with a single layer and 500 μm interspace. Representative pictures of c) SF free-standing scaffold and d) its flexibility.*

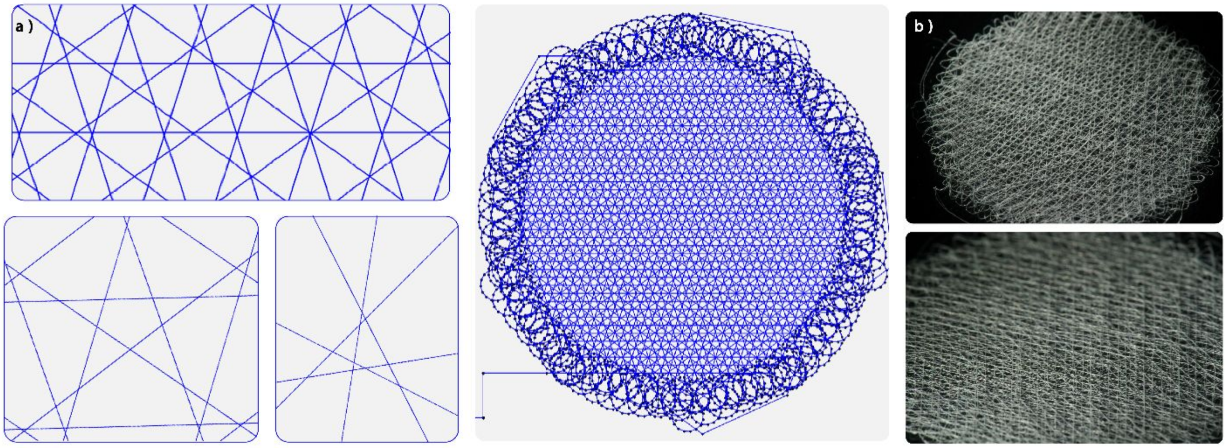

Figure S6. *a) G-Code S8 graphical representation, and zoom on most representative spots, b) optical images of complex SF scaffold with 25 layers (G-code S8).*

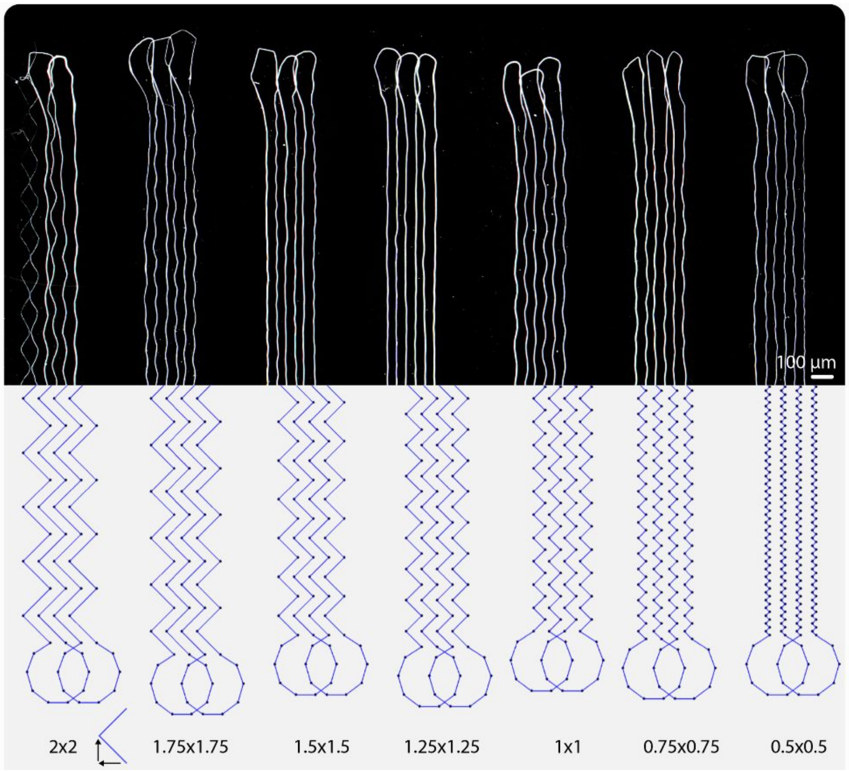

Figure S7. *Sinusoidal fibers optical image and G-code graphical representation.*
